# Supplementary material for: Identification of photoperiod-induced specific miRNAs in the adrenal glands of Sunite sheep (Ovis aries)
Source: Front Vet Sci. 2022 Jul 22;9:888207. doi: 10.3389/fvets.2022.888207 (PMC9354845; doi:10.3389/fvets.2022.888207)
Supplement: Supplementary file 1 [file Data_Sheet_1.ZIP › Supplementary file/miRDeep2 file.zip!/miRDeep2 file/Know_Structure/SPLP42A3/oar-mir-543.pdf]

miRBase precursor : oar-mir-543
Total read count : 15961
oar-miR-543-5p read count : 8
oar-miR-543-3p read count : 15920
remaining reads : 33

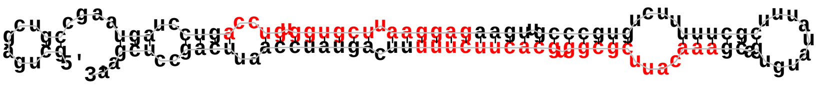

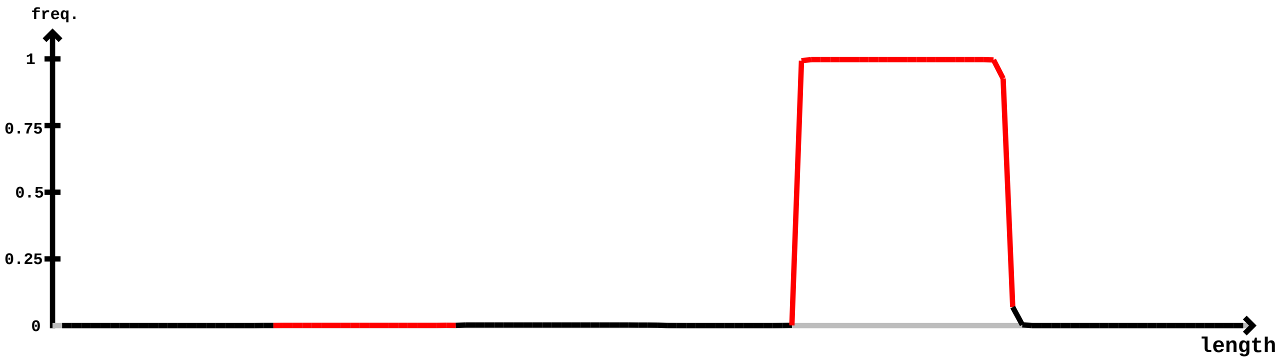

oar-miR-543-3p

oar-miR-543-5p

```
5'- gcugagcugccgaaugauccugaccuguggugcuuaaggagaaguugcccgugucuuuuucgcuuuauaugugacgaaacauucgcggugcacuucuuuuucaguauccaauucagccucgaa   -3'   exp
    ((......)).....((..((((..((.((((((.((((((((((.(((((((....((((((.........).)))))....))))).)))))))))))).))))))))..))))..))...    reads   mm
    ....................ugaccuguggugcuuaagga...................................................................................    4       0
    .....................gaccuguggugcuuaagga...................................................................................    1       0
    ......................accuguggugcuuaagga...................................................................................    3       0
    .......................................agaaguugcccgugucuuuu................................................................    4       0
    .......................................agaaguugcccgugucuuuuucg.............................................................    1       0
    .......................................agaaguugcccgugucuuuuucgc............................................................    1       0
    ........................................gaaguugcccgugucuuuuucg.............................................................    3       0
    .........................................aaguugcccgugucuuuuu...............................................................    1       0
    .........................................aaguugcccgugucuuuuuc..............................................................    5       0
    .........................................aaguugcccgugucuuuuucg.............................................................    16      0
    .........................................aaguugcccgugucuuuuucgcu...........................................................    2       0
    ..........................................................................cgaaacauucgcggugcacuuc...........................    1       0
    ..........................................................................cgaaacauucgcggugcacuucu..........................    2       0
    ...........................................................................gaaacauucgcggugcacuucu..........................    8       0
    ............................................................................aaacauucgcggugcacu.............................    1       0
    ............................................................................aaacauucgcggugcacuuc...........................    19      0
    ............................................................................aaacauucgcggugcacuucu..........................    1099    0
    ............................................................................aaacauucgcggugcacuucuu.........................    13625   0
    ............................................................................aaacauucgcggugcacuucuuu........................    1051    0
    ............................................................................aaacauucgcggugcacuucuuuu.......................    43      0
    .............................................................................aacauucgcggugcacuucu..........................    4       0
    .............................................................................aacauucgcggugcacuucuu.........................    57      0
    .............................................................................aacauucgcggugcacuucuuu........................    8       0
    .............................................................................aacauucgcggugcacuucuuuu.......................    2       0
```
